# Safety and efficacy of Ninjin’yoeito along with iron supplementation therapy for preoperative anemia, fatigue, and anxiety in patients with gynecological disease: an open-label, single-center, randomized phase-II trial

**DOI:** 10.1186/s12905-022-01824-9

**Published:** 2022-06-14

**Authors:** Taro Yagi, Kenjiro Sawada, Mayuko Miyamoto, Yasuto Kinose, Satoshi Nakagawa, Tsuyoshi Takiuchi, Michiko Kodama, Eiji Kobayashi, Kae Hashimoto, Seiji Mabuchi, Takuji Tomimatsu, Kiyoshi Yoshino, Tadashi Kimura

**Affiliations:** 1 Department of Obstetrics and Gynecology, Osaka University Graduate School of Medicine, 2-2, Yamada-oka, Suita-city, Osaka, Japan; 2 Department of Clinical Genomics, Osaka University Graduate School of Medicine, Osaka, Japan; 3 Department of Genetic Counseling, Osaka University Hospital, Osaka, Japan; 4 Department of Gynecologic Oncology, Osaka International Cancer Institute, Osaka, Japan; 5 Department of Obstetrics and Gynecology, University of Occupational and Environmental Health, Fukuoka, Japan

**Keywords:** Anemia, Anxiety, Fatigue, Ferritin, Herbal medicine, Iron deficiency, Ninjin'yoeito

## Abstract

**Background:**

Preoperative anemia affects perioperative outcomes and often causes fatigue and psychological disorders. Therefore, anemia should be treated before a patient undergoes surgery. Ninjin’yoeito (NYT), a Japanese Kampo medicine composed of ginseng and Japanese angelica root with the other 10 herbs, is administered for anemia, fatigue and anxiety; however, there are a few reports that have prospectively examined the effects of NYT before surgery for gynecological diseases. Hence, we tended to investigate its efficacy and safety.

**Methods:**

In this open-label randomized trial, women with gynecological diseases accompanied by preoperative anemia (defined as < 11.0 g/dL Hemoglobin [Hb]) were randomly assigned (1:1) into the iron supplementation and NYT groups. Patients of the iron supplementation group and the NYT group received 100 mg/day iron supplementation with and without NYT (7.5 g/day) for at least 10 days before surgery. The primary endpoint was improvement in Hb levels before and after treatment, and Cancer Fatigue Scale (CFS) and Visual Analogue Scale for Anxiety (VAS-A) scores between groups. Statistical analyses were performed with Wilcoxon signed rank test, Wilcoxon rank sum test, and Fisher’s exact test as appropriate.

**Results:**

Forty patients were enrolled of whom 30 patients were finally analyzed after allocating 15 to each group. There was no difference in the characteristics between both groups. Hb significantly increased in both groups (iron supplementation group, 9.9 ± 0.8 g/dL vs. 11.9 ± 1.6 g/dL; NYT group, 9.8 ± 1.0 g/dL vs. 12.0 ± 1.0 g/dL); the difference in the elevations in Hb between both groups was statistically insignificant (*P* = 0.72). Contrarily, CFS (17.9 ± 10.2 vs. 8.1 ± 5.2) and VAS-A (56 mm (50–70) vs. 23 mm (6–48)) scores were significantly decreased only in the NYT group and these changes were greater in the NYT group (∆CFS, *P* = 0.015; ∆VAS-A, *P* = 0.014). Liver dysfunction occurred in one patient of the NYT group.

**Conclusions:**

For treating preoperative anemia in women with gynecological conditions, NYT administration along with iron supplementation safely and efficiently improved the preoperative fatigue and anxiety in addition to the recovery from anemia.

*Trial registration*: jRCT1051190012 (28/April/2019, retrospectively registered).

**Supplementary Information:**

The online version contains supplementary material available at 10.1186/s12905-022-01824-9.

## Background

World Health Organization estimates that one-third of all women of reproductive age are anemic [1], and women of reproductive age are most likely to be affected by iron deficiency, which is responsible for approximately 51% of global cases of anemia [2]. In 18 large observational studies encompassing over 650,000 surgical patients, the mean prevalence of pre-operative anemia was around 35%, varying between 10.5% and 47.9% [3]. In a study by Richards T, et al., among 12,836 patients undergoing gynecological surgery, the prevalence of preoperative anemia was 23.9% (95% CI 23.2–24.7) [4]. Representative gynecological diseases such as polyp, adenomyosis, uterine fibroids and malignancies often cause abnormal uterine bleeding (AUB), and on average, 30% of women globally with AUB are anemic [2]. Furthermore, preoperative anemia can affect surgical outcomes and may contribute to postoperative complications such as cardiac disease, respiratory disease, systemic sepsis, venous thrombosis, and major bleeding [4]. In a study by Musallam KM et al., the 30-day postoperative mortality rate among non-cardiac surgery patients was significantly higher in the anemic group than in a non-anemic group [5] and preoperative anemia was concluded as a risk factor for prolonged postoperative hospitalization, readmission, and increased rates of postoperative complications in patients who underwent laparoscopic hysterectomy [6]. Further, anxiety and depressed mood occurred in 25–80% of patients scheduled for surgery, and younger age, female sex, shortened sleeping period, undergoing the first surgery, history of cancer, undergoing gynecological or cosmetic surgery, and general anesthesia are reported to be risk factors [7]. Such mental states have also been associated with unspecified complaints including insomnia and fatigue [8], which eventually lead to poorer surgical outcomes [9, 10], increased postoperative complications [11, 12], and greater postoperative pain [13]. Iron deficiency anemia has been shown to be associated with anxiety disorders, depression, sleep disorders, adverse psychomotor function disorders, reduced physiological capacity, and delayed socioemotional development [14]. For instance, iron deficiency affects the dopaminergic neurotransmission in the brain, which can cause depression [15]. In a rodent model, tyrosine from protein-rich foods was converted to dopamine only when iron was present in the cells [16], and lack of dopamine can cause depression, anxiety, and even restless leg syndrome [17]. Given that approximately 50% of patients scheduled for gynecological disease surgery are anemic and that most are case of iron deficiency, their immediate treatment by iron supplementation can improve the physical and psychological states, even postoperatively.

Ninjin'yoeito (NYT), a Japanese Kampo medicine, has been derived from the ancient Chinese medical literature and is composed of 12 herbs [18], namely, ginseng, Japanese angelica root, peony root, rehmannia root, atractylodes rhizome, poria sclerotium, cinnamon bark, astragalus root, citrus unshiu peel, polygala root, schisandra fruit, glycyrrhiza. As it translates to "ginseng nutritive decoction," based on its main ingredient, it is known to improve the nutritional status and used as herbal medicine for symptomatic treatment of "post-illness weakness, fatigue, loss of appetite, night sweats, cold extremities, and anemia." Previous clinical and preclinical studies concluded on its effectiveness in anemia [19, 20], fatigue [21–27], and psychological conditions, namely anxiety and depression, as well as lethargy and apathy associated with Alzheimer's and Parkinson's diseases [28–32]. However, very little prospective research has been performed to investigate the effects of administering NYT in patients having preoperative anemia with unspecified symptoms, especially for gynecological diseases.

Therefore, this study explored the effectiveness and safety of administering NYT in addition to iron supplementation in patients with gynecological diseases with preoperative anemia. The study compared two groups of patients who were randomly assigned (1:1) to receive iron supplementation or iron supplementation with NYT.

## Methods

### Study design and ethics approval

This was a non-blinded, randomized controlled trial conducted at the Osaka University Hospital (Suita, Osaka, Japan). Efficacy and safety were evaluated in two groups, one receiving only 100 mg/day iron supplementation (sodium ferrous citrate) and the other NYT group receiving 7.5 g/day of NYT extract granules along with iron supplementation (100 mg/day sodium ferrous citrate). This study was conducted in accordance with the Declaration of Helsinki [33] and the Clinical Trials Act stipulated by the Ministry of Health, Labour and Welfare in Japan [34], and CONSORT 2010 statement [35]. It was approved by the certificated review board of Osaka University Hospital on 13/10/2017 (N18032), and later registered in jRCT1051190012 on 28/04 /2019. All methods were performed in accordance with the relevant guidelines and regulations determined by Osaka University Research Ethics Committee, and written informed consent was obtained from each participant. The study protocol is available from https://jrct.niph.go.jp/en-latest-detail/jRCT1051190012. The first participant was registered on 01/11/2017.

### Subjects

Women aged 20 or older who were scheduled for gynecological surgery at the Department of Obstetrics and Gynecology under the Osaka University Hospital between October 2017 and August 2021were enrolled into this study. All subjects had preoperative anemia based on the WHO criteria of hemoglobin (Hb) < 11.0 g/dL without other non-gynecological diseases that could cause anemia [36]. The exclusion criteria were as follows: patients suspected with non-iron deficiency anemia based on their medical history findings and blood test data; those receiving other Kampo formulas or herbal preparations; patient who had treated with other Kampo formulas within 2 weeks before this study started; those diagnosed with depression, aldosteronism, myopathy, hypokalemia, or liver dysfunction (AST ≥ 80 IU/L or ALT ≥ 80 IU/L) or those suspected with these conditions; and patients otherwise determined as ineligible by a doctor. Patients who consented to participate were randomly assigned to the iron supplement only group (iron supplementation group) or the iron plus NYT group (NYT group) at a 1:1 ratio using a random number table (MM administrated the random number table).

### Interventions

All details of NYT attachment, ingredients, and formulations of the constituent herbs are listed in STORK [37]. Two treatments were administered: sodium ferrous citrate (oral Ferromia 50 mg tablets, Eisai Co., Tokyo, Japan) 100 mg/day, known as the iron supplementation group (control group); the other group was the NYT group (intervention group), which received sodium ferrous citrate 100 mg/day plus NYT granules (KB-108, Kracie Pharma Ltd., Tokyo, Japan) 7.5 g/day. This drug is under the insurance coverage and the dose used is determined by Pharmaceuticals and Medical Devices Agency in Japan as previously reported [29, 38].

After written informed consent was obtained from each participant, peripheral blood was collected and complete blood count including red blood cell count, Hemoglobin (Hb), Hematocrit (Ht), serum iron, and ferritin were analyzed. Thereafter, the pre-decided interventions were prescribed to the patients on outpatient clinic visits. At admission for surgery, each laboratory parameter was re-analyzed and the change rates for each parameter was calculated. If patients required emergency surgeries and the treatments had been administered for less than 10 days, then those patients were excluded from this trial. Additionally, the participants were instructed to answer the questionnaires consisting of the Cancer Fatigue Scale (CFS), visual analogue scale for anxiety (VAS-A), and Pittsburgh sleep quality index (PSQI) at the entry and at admission. [39–43]. CFS is a 15-item scale measuring the severity of fatigue that consists of three domains: physical, affective, and cognitive. Higher total scores (overall fatigue) indicate more severe fatigue [39, Additional file 1: Table S1]. VAS-A is scored on a straight line 100-mm long, with the left end (0 mm) representing no anxiety and the right end (100 mm) representing most severe anxiety. This is the most useful index for assessing the perioperative anxiety [40, 41]. PSQI is an 18-item questionnaire having 7 domains: sleep quality, sleep duration, sleep latency, sleep efficiency, sleep disturbances, use of sleeping medication, daytime sleepiness, and other dysfunctionality [42, Additional file 2: Table S2]. Higher scores indicate more severe sleep disturbances. It is considered a highly versatile sleep index [43].

### Outcomes

The primary outcomes were the changes in Hb, CFS, and VAS-A levels before and after treatment. The secondary outcomes were safety and changes in serum iron, ferritin, and PSQI before and after treatment.

### Sample size calculation

Referring to the previous reports [29, 38] comparing the effects of iron supplementation alone and iron supplementation in combination with NYT on anemia, the change in mean Hb after the treatment was an increase of 0.7 g/dl in the iron supplementation alone group and that of 1.2 g/dl in the combination group. Assuming that the difference between the means of the two groups is 0.5 and the standard deviation (common to both groups) is 0.5, and assuming an alpha error rate of 5% and a beta error rate of 20%, 17 cases in each group are needed. Therefore, we set the target number of cases to 40 to meet the required number of cases.

### Statistical analysis

JMP 16.0.0 (SAS Institute Inc., Cary, NC, USA) was used for statistical analysis. We compared the baseline characteristics of patients with the Wilcoxon rank sum test and Fisher’s exact test. The changes in Hb, CFS, and VAS-A values before and after treatment in each group were statistically analyzed with the Wilcoxon signed rank test. Wilcoxon rank sum test was performed when comparing the amount of change in those before and after treatment between control and intervention group. P-value of < 0.05 was considered for statistical significance. Normally distributed values are expressed as mean ± standard deviation and those with non-normal distributions are expressed as median and interquartile ranges.

## Results

**Fig. 1:**
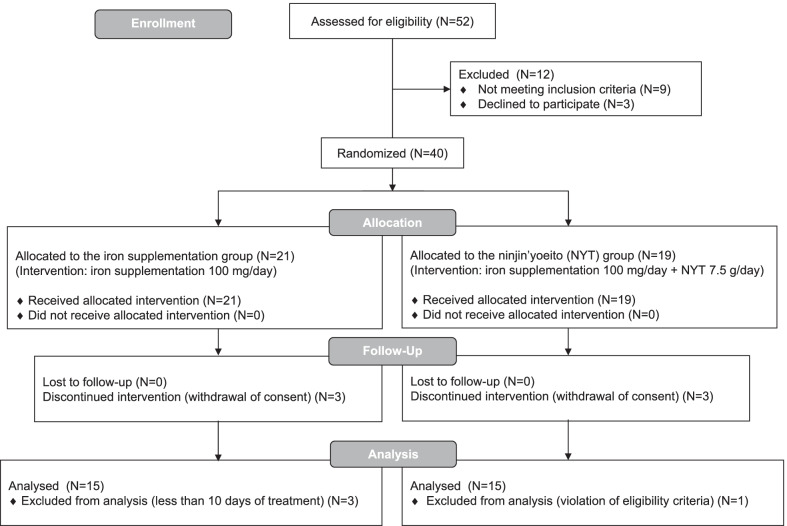
CONSORT Flow Diagram of the participants in this study

Forty patients enrolled into the study from October 2017 to August 2021, 6 patients withdrew their consent; 3 patients were excluded because their treatment period was < 10 days; 1 patient was excluded because of violation of eligibility criteria. Finally, 15 patients in the iron supplementation group and 15 patients in the NYT group were analyzed (Fig. 1). Among those, 12 patients with uterine fibroid underwent surgeries due to menorrhagia. Nine patients underwent surgeries due to the treatment of malignant cancers. Seven patients underwent surgeries due to benign ovarian tumors. One patient with multiple endometrial polyps underwent surgery due to menorrhagia. One patient with uterine prolapse underwent surgery due to worsening her symptom. Mean Hb of the 30 cases was 9.8 ± 0.9 g/dL, mean Ht was 32.5% ± 2.0%, mean corpuscular volume was 79.1 ± 8.4 fL, and mean corpuscular hemoglobin concentration was 30.3% ± 1.7%. Serum iron and ferritin levels were measured in 10 cases before starting treatment. The median serum iron concentration was 15 (14–26) µg/dL with all cases below our hospital's reference range (41–127 µg/dL). The median ferritin level was 3 (2–10) ng/mL with none being higher than our institutional reference range (4–123 ng/mL). No patient had non-gynecological diseases that could have induced anemia, and thus all patients were treated based on a diagnosis of iron deficiency anemia.

In the iron supplementation group, the mean age was 45.1 ± 7.7 years, body mass index (BMI) was 21.4 ± 2.6 kg/m^2^, and the length of administration was 39.7 ± 23.0 days. 14 patients were non-menopausal and 5 received hormone therapy such as GnRH antagonist treatment during the study period. Uterine fibroid was the most common in the iron supplementation group, as 7 patients had uterine fibroid (47%), while there were 5 patients with malignant disease (33%). In contrast, the NYT group had a mean age of 41.1 ± 8.3 years with a mean BMI of 22.6 ± 2.7 kg/m^2^, and length of administration was 39.9 ± 20.3 days. All patients were non-menopausal and 3 received hormone therapy during the study period, respectively. Uterine fibroid was again most common in the NYT group, as 5 patients had uterine fibroids (33%), while 4 patients had malignant disease (27%). None of these values showed significant between-group differences, neither of the baseline characteristics had significant differences between the groups (Table 1).

**Table 1:** Baseline characteristics of the included patients

		Iron supplementation group (N = 15)	NYT group (N = 15)	*P* value
Age-years		45.1 ± 7.7	41.1 ± 8.3	0.52
Body mass index-kg/m^2^		21.4 ± 2.6	22.6 ± 2.7	0.27
Duration of intervention (IQR)-days		39.7 ± 23.0 (23, 50)	39.9 ± 20.3 (21, 58)	0.88
Disease-N (%)				1
Benign		10 (67)	11 (73)	
Malignant		5 (33)	4 (27)	
Length of surgery-min		221 ± 158	190 ± 152	0.76
Menstruation-N (%)				1
+		14 (93)	15 (100)	
−		1 (7)	0 (0)	
Hormonal therapy-N (%)				0.68
+		5 (33)	3 (20)	
−		10 (67)	12 (80)	
Hemoglobin-g/dL		9.9 ± 0.8	9.8 ± 1.0	0.59
Hematocrit-%		32.3 ± 2.1	32.7 ± 2.1	0.77
Median serum iron level (IQR)-µg/dL		27 (14, 84) ^$^	32 (15, 146) ^#^	0.88
Median ferritin (IQR)-ng/mL		10 (5, 22) ^$^	4 (3, 10) ^#^	0.18
CFS				
Physical		5.9 ± 5.1	7.1 ± 5.4	0.4
Affective		6.5 ± 3.0	7.9 ± 4.1	0.38
Cognitive		3.5 ± 3.7	2.8 ± 2.8	0.92
Total		15.9 ± 9.3	17.9 ± 10.2	0.66
Median VAS-A (IQR)-mm		50 (8, 65)	56 (50, 70)	0.38
Median PSQI (IQR)		4 (3, 5)	5 (3, 7)	0.37

Plus-minus values are mean ± standard deviation; analyzed with Wilcoxon rank sum test or Fisher’s exact test

*CFS* cancer fatigue scale, *VAS-A* visual analogue scale for anxiety, *PSQI* Pittsburgh sleep quality index, *IQR* interquartile range

$, N = 9; #, N = 8

Comparing the outcomes in the iron supplementation group before and after treatment, significant increases were observed in Hb (9.9 ± 0.8 g/dL vs. 11.9 ± 1.6 g/dL) and Ht (32.3% ± 2.1% vs. 37.1% ± 4.3%), but no significant differences were observed in CFS and VAS-A scores and serum iron, ferritin, or PSQI values. Conversely, when the NYT group data from before and after treatment were compared, the Hb (9.8 ± 1.0 g/dL vs. 12.0 ± 1.0 g/dL), Ht (32.7% ± 2.1% vs. 38.2% ± 3.3%), and ferritin (median 4 ng/mL (3–10) compared with median of 19 ng/mL (10–26) significantly increased, while the CFS subscales (physical fatigue 7.1 ± 5.4 vs. 2.5 ± 2.4, affective fatigue 7.9 ± 4.1 vs. 4.9 ± 3.4, cognitive fatigue 2.8 ± 2.8 vs. 0.7 ± 1.0, overall fatigue 17.9 ± 10.2 vs. 8.1 ± 5.2) and VAS-A (56 (50–70) mm compared with median of 23 (6–48) mm) significantly decreased. Moreover, the serum iron or PSQI values from before to after treatment had no significant differences (Table 2).

**Table 2:** Each effectiveness of Iron supplementation or Iron supplementation with Ninjin’yoeito for treating perioperative anemia, fatigue and anxiety

		Iron supplementation group (N = 15)			NYT group (N = 15)		
		Pre-treatment	Post-treatment	*P* value	Pre-treatment	Post-treatment	*P* value
Hemoglobin-g/dL		9.9 ± 0.8	11.9 ± 1.6	0.0005	9.8 ± 1.0	12.0 ± 1.0	0.0001
Hematocrit-%		32.3 ± 2.1	37.1 ± 4.3	0.001	32.7 ± 2.1	38.2 ± 3.3	0.0002
Median serum iron level (IQR)-µg/dL		27 (14, 84) ^$^	125 (88, 214)^~^	0.055	32 (15, 146) ^#^	100 (81, 205) ^|^	0.38
Median ferritin (IQR)-ng/mL		10 (5, 22) ^$^	26 (16, 31) ^~^	0.063	4 (3, 10) ^#^	19 (10, 26) ^|^	0.0078
CFS							
Physical		5.9 ± 5.1	4.1 ± 4.1	0.23	7.1 ± 5.4	2.5 ± 2.4	0.0005
Affective		6.5 ± 3.0	6.5 ± 2.5	0.96	7.9 ± 4.1	4.9 ± 3.4	0.025
Cognitive		3.5 ± 3.7	2.0 ± 2.3	0.094	2.8 ± 2.8	0.7 ± 1.0	0.002
Total		15.9 ± 9.3	12.5 ± 6.5	0.1	17.9 ± 10.2	8.1 ± 5.2	0.0004
Median VAS-A (IQR)-mm		50 (8, 65)	32 (13, 57)	0.2	56 (50, 70)	23 (6, 48)	0.0009
Median PSQI (IQR)		4 (3, 5)	4 (3, 6)	1	5 (3, 7)	4 (3, 5)	0.15

Plus-minus values are mean ± standard deviation; analyzed with Wilcoxon signed rank test

*CFS* cancer fatigue scale, *VAS-A* visual analogue scale for anxiety, *PSQI* Pittsburgh sleep quality index, *IQR* interquartile range

$, N = 9; #, N = 8; ~ , N = 13; |, N = 11

**Fig. 2:**
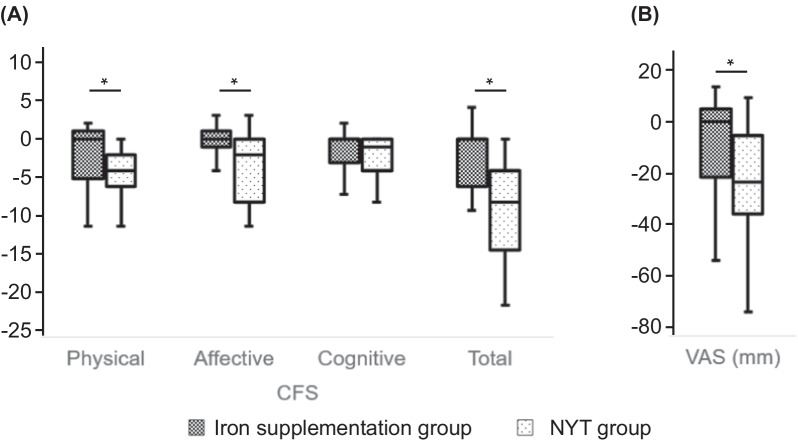
Comparison of the treatment effectiveness between the iron supplementation group and the NYT group. **A** Plot of the Cancer Fatigue Scale (CFS) scores; **B** the Visual Analogue Scale for Anxiety (VAS-A) scores, examining the treatment effect between the two groups; NYT, Ninjin’yoeito; *P < 0.05

The differences in the changes in Hb, Ht, cognitive fatigue, serum iron, ferritin, or PSQI between the iron supplementation group and NYT group were non-significant. Regarding the CFS domain values, the NYT group showed significant reduction in terms of physical fatigue (− 1.8 ± 3.8 vs. − 4.6 ± 4.2), affective fatigue (− 0.1 ± 1.9 vs. − 3.1 ± 4.4), and overall fatigue (− 3.5 ± 7.2 vs. − 9.7 ± 8.2; Fig. 2A). The NYT group also showed a significant decline in the VAS-A score (0 mm (− 20–5) versus − 24 mm (− 35 to − 4); Fig. 2B). Lastly, liver dysfunction was observed in 1 patient in the NYT group during this period.

## Discussion

This study analyzed the effects of combining NYT with iron supplementation to treat preoperative anemia and its associated physical and psychological symptoms. The Hb and Ht levels increased significantly in both groups. Further, the NYT group exhibited significant improvements in the physical, affective, and overall CFS fatigue indices and the VAS-A values. The improvements were statistically significant when compared with those in the iron supplementation group. Fatigue and anxiety are strongly associated with anemia, which suggests the usefulness of combining NYT with iron supplements to treat anemia before patients are scheduled for surgical treatment of gynecological diseases.

Although NYT was reported to promote myelopoiesis by accelerating the differentiation of pluripotent hematopoietic stem cells [44–46], which suggests its clinical efficacy for anemia [19, 20], the results of this study showed no statistically significant differences in anemia improvement between the respective groups. Because there was only 1 severe case with Hb < 8 g/dL, iron supplementation alone may have rapidly improved their iron deficiency anemia.

Regarding fatigue and anxiety as analyzed by the CFS and VAS-A values, significant improvements were not observed in the iron supplementation group, while fatigue significantly improved and anxiety disappeared in the NYT group. Patients who will undergo surgery experience emotional distress in the form of anxiety about postoperative pain, complications, and the results of the procedure, as well as concern from their family. They also experience stress from factors including the unfamiliar medical environment and unusual schedule. Stress responses against such situations are triggered by various chemical mediators such as neurotransmitters (e.g. noradrenaline and dopamine), hormones (e.g. corticoids) and cytokines [47, 48]. This can result in not only anxiety but also various unspecified complaints such as fatigue, insomnia, and loss of appetite [7, 8, 49, 50]. It has also been reported that, in systemic inflammatory diseases such as cancer, an increase in inflammatory cytokines from the immune response can lead to deterioration of the mental state [50]. NYT has been suggested to improve the appetite via dopamine and NPY pathway [51–53] and to inhibit corticosterone by inhibiting NO synthesis [30]. In addition, schisandra fruit and citrus unshiu peel have been reported to have anti-anxiety effects [28, 54, 55] and ginseng has been reported to have anti-anxiety and anti-fatigue effects [56, 57]. All of these are ingredients of NYT. Other ingredients (Japanese angelica root, cinnamon bark, polygala root, glycyrrhiza) have also been reported to have anti-inflammatory effects [58], and it is thought that, in combination, these ingredients act to improve fatigue and anxiety. In general, in order to address preoperative anxiety and unspecified complaints, the effects of anti-anxiety drugs are limited and there is no established drug therapy [7, 59]. Therefore, the effectiveness of NYT in treating preoperative fatigue and anxiety in this study could provide an opportunity for overcoming these conditions.

In routine clinical practice, anti-anxiety drugs are considered as options for patients experiencing preoperative anxiety and other unspecified complaints. However, such drugs have side effects including dependence, drowsiness, dizziness, and lassitude [60], but the use of NYT has not been associated with side effects such as dependence and drowsiness [27]. In that sense, the effectiveness of NYT in treating preoperative fatigue and anxiety in this study could provide an opportunity for overcoming these conditions. Especially, it may be safe even for elderly patients, given that the only side effect reported in our cohort was one case of mild liver dysfunction in the NYT group, which improved quickly after NYT was paused. There were no complications that affected the treatment of gynecological conditions, such as by prolonging hospitalizations, which indicates that the formula can be used safely. Moreover, NYT has been suggested to be effective not only perioperatively but also as a supportive therapy for managing complications associated with radiation and chemotherapy [23–25, 44, 61]. As it can provide a comprehensive treatment at any stage of patients with gynecological diseases, it has been concluded as a promising Kampo formula.

In this study, one participant in NYT group presented live dysfunction. However, her liver dysfunction was very mild and she recovered soon after the discontinuation of the drugs she took. Since she took not only iron supplementation and NYT but also relugolix and loxoprofen sodium hydrate, it was difficult to determine which drug contributed to the elevated liver enzyme. A utilization study regarding the safety and effectiveness of ninjin'yoeito was reported [27]. Among 808 elderly patients with whom ninjin'yoeito was treated, adverse reactions were reported in 25 patients (3.1%), most of whom had gastrointestinal disorders (2.1%) and none presented liver dysfunction. Thus, it appears to be reasonable to consider that her liver dysfunction is not solely caused by NYT administration.

The limitations of this study include the lack of blinding with a placebo, since it was not possible to provide placebo compounds for ethical and financial reasons. Second, the sample size was small. Third, this study was registered initially at University hospital Medical Information (UMIN) locally available in Japan on 01/11/2017 (UMIN000029525, https://center6.umin.ac.jp/cgi-openbin/ctr/ctr_view.cgi?recptno=R000031244). However, with the enactment of the Clinical Trials Act stipulated by the Ministry of Health, Labour and Welfare in Japan on 01/04/2018, Japan Registry of Clinical Trials (jRCT) was newly established and the function of UMIN was transferred to jRCT. Hence, this study appears to be retrospectively registered. Forth, patients with a variety of gynecological diseases were included in this study. The level of fatigue and anxiety might differ between those with benign tumors and those with malignant tumors, and such potential observational bias could not be eliminated due to the small sample size. Therefore, a double-blind randomized controlled trial with a larger sample size needs to be conducted in the future. Further, although our analysis was limited to the preoperative therapeutic effects, it is thought that NYT may help alleviate the physical and psychological symptoms of patients after surgery. Therefore, in the future we need to investigate whether NYT can contribute to improving the postoperative outcomes.

## Conclusions

The combined use of NYT and iron supplements for anemia before surgery due to gynecological diseases was able to improve the anemia and significantly alleviate their fatigue and anxiety as well. This combination therapy is an effective treatment option in gynecological care not only because improving anemia reduces the frequency of postoperative complications, but also because it can help improve fatigue and anxiety, which are directly linked to postoperative recovery.

## Supplementary Information

**Additional file 1: Supplemental Table 1. Questionnaires of The Cancer Fatigue Scale.** The Cancer Fatigue Scale is a 15-item scale measuring the severity of fatigue that consists of three domains: physical, affective, and cognitive. Higher total scores (overall fatigue) indicate more severe fatigue [39].

**Additional file 2: Supplemental Table 2. Questionnaires of The Pittsburgh Sleep Quality Index.** The Pittsburgh Sleep Quality Index is an 18-item questionnaire having 7 domains: sleep quality, sleep duration, sleep latency, sleep efficiency, sleep disturbances, use of sleeping medication, daytime sleepiness, and other dysfunctionality. Higher scores indicate more severe sleep disturbances [42].

## Data Availability

The datasets used and/or analyzed during the current study are available from the corresponding author on reasonable request.

## Abbreviations

NYT: Ninjin'yoeito
NPY: Neuropeptide Y
PSQI: Pittsburgh sleep quality index
CFS: Cancer Fatigue Scale
VAS-A: Visual analogue scale
